# Cardiac Autonomic Modulation in Response to Muscle Fatigue and Sex Differences During Consecutive Competition Periods in Young Swimmers: A Longitudinal Study

**DOI:** 10.3389/fphys.2021.769085

**Published:** 2021-11-18

**Authors:** Matías Castillo-Aguilar, Pablo Valdés-Badilla, Tomás Herrera-Valenzuela, Eduardo Guzmán-Muñoz, Pedro Delgado-Floody, David Cristóbal Andrade, Michele M. Moraes, Rosa M. E. Arantes, Cristian Núñez-Espinosa

**Affiliations:** ^1^Kinesiology Department, University of Magallanes, Punta Arenas, Chile; ^2^Austral Integrative Neurophysiology Group, CADI-UMAG, Punta Arenas, Chile; ^3^Department of Physical Activity Sciences, Faculty of Education Sciences, Universidad Católica del Maule, Talca, Chile; ^4^Carrera de Entrenador Deportivo, Escuela de Educación, Universidad Viña del Mar, Viña del Mar, Chile; ^5^Department of Sports Sciences and Physical Activity, Faculty of Health, Universidad Santo Tomás (UST), Santiago, Chile; ^6^Department of Physical Activity, Sports and Health Sciences, Faculty of Medical Sciences, Universidad de Santiago de Chile (USACH), Santiago, Chile; ^7^Escuela de Kinesiología, Facultad de Salud, Universidad Santo Tomás, Santiago, Chile; ^8^Department of Physical Education, Sports and Recreation, Universidad de La Frontera, Temuco, Chile; ^9^Centro de Investigación en Fisiología y Medicina de Altura (MedAlt), Facultad de Ciencias de la Salud, Universidad de Antofagasta, Antofagasta, Chile; ^10^Department of Pathology, Institute of Biological Sciences, Universidade Federal de Minas Gerais, Belo Horizonte, Brazil; ^11^Faculty of Medicine, Center for Newborn Screening and Genetics Diagnosis, Universidade Federal de Minas Gerais (NUPAD-FM/UFMG), Belo Horizonte, Brazil; ^12^School of Medicine, University of Magallanes, Punta Arenas, Chile; ^13^Interuniversity Center for Healthy Aging, Chile

**Keywords:** arterial pressure, autonomic nervous system, heart rate variability, physical exertion, swimming

## Abstract

**Objective:** To study the differences in cardiac autonomic modulation in response to muscle fatigue caused by high-intensity exercise during two consecutive competition periods in young swimmers.

**Methods:** Twenty-six competitive swimmers, selected by their training volume, were separated in two groups, females (*n* = 12 [46%], age: 13.5 ± 1.4 years) and males (*n* = 14 [54%], age: 13.9 ± 1.7 years), aged between 10 and 16 years, were evaluated five times as follow: (i) 21 days before the first competition (t-0); (ii) two days before (t-1; t-3); and (iii) two days after (t-2; t-4) of the first and second competitions. Morphological measurements (body mass, percentage of total body fat and height), blood pressure, power, and resting heart rate variability (RR with Polar band) were recorded before and after Wingate test at each time.

**Results:** Body fat was higher in females compared to males. However, no differences were found in other morphological parameters. An intra-subject analysis grouped by sex in cardiovascular parameters shows longitudinal variations in systolic pressure and mean pressure among females. Additionally, females depicted higher, very low frequency (VLF, which is intrinsically generated by the heart and strongly associated with emotional stress) after physical fatigue compared to males at t-1. Further, before the competition, the high frequency (HF) component of HRV (parasympathetic drive) was higher in males than females at t-0 and t-4.

**Conclusion:** Our data revealed that males displayed greater parasympathetic reactivity after an anaerobic muscle fatigue test during their competition periods. Contrarily, females had a less cardiac autonomic modulation when comparing the pre-post Wingate test after two consecutive competition periods.

## Introduction

Young swimmer athletes display different autonomic nervous system (ANS) modulation of the heart compared to non-trained control subjects (Arce-Álvarez et al., 2021). Considering the amount of research in this area, cardiac autonomic modulation by heart rate variability (HRV) has emerged as a highly effective tool to access neurophysiological adaptations demanded at different athlete’s training moments (Clemente-Suárez et al., 2015; Arce-Álvarez et al., 2021). Of note, it has been relevant to the point that it is capable of overtraining states (Kamandulis et al., 2020) and estimating athletic performance (Chalencon et al., 2015). Notably, there are several techniques and calculations focused on determining autonomic modulation; nevertheless, most are invasive maneuvers, which reduce their practical use (i.e., sports activities). Thus, it has been proposed that HRV calculated from signals obtained from portable devices could reflex autonomic modulation of the heart with high reliability (Georgiou et al., 2018).

Interestingly, it is very well known that during training and competition, muscle fatigue is generated, and it has been proposed that this fatigue could play a pivotal role in the cardiac autonomic response of an athlete (Buchheit et al., 2011; Mayo et al., 2016). Indeed, low-intensity training for long periods and short high-intensity exercises with long recovery periods seems to be a stronger modulator of cardiac autonomic response swimmers’ athletes during training (Clemente-Suárez and Arroyo-Toledo, 2018; Pla et al., 2019). In this sense, recovery of parasympathetic discharge is modulated by training intensity, with delay for recovery ANS/HRV in athletes exercised at and above the first ventilatory threshold (Seiler et al., 2007).

Muscle fatigue favors parasympathetic withdrawal, which could affect the recovery period; however, it is expected that this phenomenon will attenuate with adequate rest and periodization, not affecting the athlete’s recovery. Thus, adequate rest and periodization promote the improvement of the neurophysiological during the tapering period, allowing the swimmer a better adaptation when competing (Flatt et al., 2017). Of note, during the competition period and/or high physical demand, swimmers’ physical preparation times can change, which could play an important role in the cardiac autonomic response, affecting the recovery periods and consequently physiological adaptations promoted by the training (Edmonds et al., 2015). In addition, the presence of differences related to age and sex in heart rate variability despite maintaining an adequate level of physical activity (based on accelerometers) can be observed at these ages (Spina et al., 2019); however, there is no evidence according to differences between females and males about the cardiac autonomic response to muscle fatigue during the competitive period.

Sex determines differences in the biological organization at all levels (Holdcroft, 2007), including cardiac modulatory balance, which has been related to a greater tone in the autonomic vagal branch in females and a greater tone in the sympathetic branch in males (Spina et al., 2019). As effort intensity also influences HRV recovery, it is noteworthy that females have an advantage in resistance to fatigue during submaximal contractions, with less impairment in neuromuscular activation after strenuous exercise; however, this advantage diminishes as the intensity of contractions increases (Azevedo et al., 2021). Therefore, considering the differences in cardiac autonomic modulation and resistance to fatigue between males and females, muscle fatigue during a competitive period may influence cardiac autonomic control in a sex-specific manner during a competitive period in swimming athletes.

At present, there is no conclusive evidence about how cardiac autonomic response could be affected during consecutive competition periods, which could affect more young swimmers who are faced with physiological and anthropometric changes typical of age (Tanina et al., 2017; Weinstein et al., 2018). Also, as differences in athletic performance of males and females begin at the age of 12–13 years (Handelsman, 2017), cardiovascular autonomic adaptation over a training season may diverge between the sexes of young swimmers. Then, we aimed to evaluate the differences in cardiac autonomic modulation through non-invasive measures in response to muscle fatigue caused by high-intensity exercise during two consecutive competition periods in young swimmers. We hypothesized that the muscular fatigue by high-intensity physical exercise during consecutive competition periods generates a cardiac autonomic response that differs according to the sex of the swimmer.

## Materials and Methods

### Design

**FIGURE 1:**
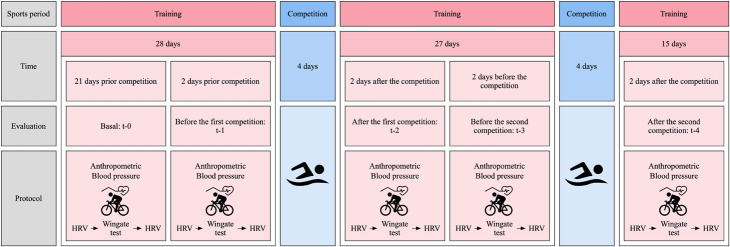
Study design and evaluations were carried out. Heart rate variability (HRV) was evaluated before and after the anaerobic physical fatigue test (Wingate test).

A descriptive, comparative, longitudinal, and quantitative approach was used. The participants were selected through intentional non-probabilistic sampling, distributed between females (*n* = 12) and males (*n* = 14). Athletes were monitored during two consecutive dates of the Patagonian International Circuit. The swimmers were observed five times. The first evaluation was carried out 21 days before the first competition (t-0). The four remaining assessments were carried out two days before (t-1; t-3; “pre-competition”) and two days after (t-2; t-4; “post-competition”) the first and second competitions. In each experimental time, anthropometric parameters (body weight, height, and percentage of body fat), cardiovascular (blood pressure), as well as HRV were measured: before and after the anaerobic muscle fatigue test (Wingate test). The study design is represented in Figure 1.

### Participants

Twenty-six competitive young swimmers of national and international competition level were recruited to participate in this study (age: 13.7 ± 1.5 years; height: 1.62 ± 0.07 m; body mass: 56.5 ± 10.5 kg; body fat: 24.4 ± 3.2%) and separate on females (age: 13.5 ± 1.4 years; height: 1.58 ± 0.03 m; body mass: 56.3 ± 9.1 kg; body fat: 30.3 ± 3.5%) and males (age: 13.9 ± 1.7 years; height: 1.65 ± 0.08 m; body mass: 56.8 ± 12.1 kg; body fat: 18.4 ± 3.6%).

The swimmers were recruited from the Fiscal Gymnasium of the Magallanes and Chilean Antarctic region. The inclusion criterion was to have a minimum of 3 years in competitive swimming, train at least six times per week, and have at least 14 h per week of training. Exclusion criteria were: take any supplements or medications that could affect the heart rate, suffer musculoskeletal injuries in the last three months, presence of pain at the time of performing the evaluations. No participants met the exclusion criteria. The volunteers and their legal guardians were informed about the objectives, procedures, responsibilities, and risks of participating in the study. Participating subjects gave their permission, and legal guardians provide informed consent before participation. The Ethics Committee approved this study of the University of Magallanes, Chile (code: N°141CEC2018), following the regulations established by the Declaration of Helsinki on ethical principles in human beings.

### Procedure

#### Training Monitoring

The swimmers were monitored during two consecutive dates of the Patagonian International Circuit.

All the swimmers were trained to perform short pool speed tests (25 m). During the training periods, before the first (between t-0 and t-1) and second (between t-2 and t-3) competition, the training routine was maintained. Throughout each pre-competitive period, a progressive decrease in training volume was carried out, starting at 7,000 m per week and ending with 3,500 m per week before the competition, progressively increasing the intensity (heart rate controlled) and giving the swimmer a longer recovery time.

To better optimize the time required for the evaluations in the athletes, measurement stations were established within the same laboratory: station 1, the athlete arrives at the laboratory, rests in a chair for 5 min, and then their blood pressure is controlled; station 2, the athlete is evaluated on his morphological measurements (approximately 10 min); Station 3, the subject remained lied on the stretcher in a supine position, and his HRV was evaluated prior to the anaerobic muscle fatigue test; station 4, the athlete is evaluated in the Wingate test, to later return to station 3, for your second HRV assessment, after the fatigue test.

#### Acute Muscle Fatigue Protocol

Among the conditions of dress, the participants were asked to wear a shirt, shorts, and footwear. Before the assessments, all participants were instructed to (a) get adequate rest the night before, sleep 8 h or more, (b) not consume stimulant drinks or medications before measurements, (c) consume ∼ 2 liters of water during the day before, (d) eat regularly without altering your diet. The participants reported to the laboratory 15 min before the test. The Wingate protocol was carried out at 22°C and 15% RH controlled by the air conditioning in a laboratory equipped for the study. For HRV, the volunteers lay down on a stretcher in the supine position, and the recording of HRV was started. During the day of testing, participants were instructed to give their maximum effort during testing.

### Assessments

#### Morphological Measures

Body mass (kg) and the total body fat (%) were assessed by bioimpedance using the Tanita BC-558 Ironman Segmental Body Composition Monitor (Tanita Ironman, Arlington Heights, IL, United States), with a concordance 89.3% compared to the Dual X-ray Absorption test using standard measurement protocols (Mialich et al., 2011; Calderón and Rodriguez-Hernandez, 2018). Height was measured by CHARDER^®^ HM230M manual height rod (Charder Electronics Co., Ltd. No.103, Guozhong Rd., Taiwan, R.O.C.). The body mass index (BMI) and the fat-free mass index (FFMI) were calculated as follow, BMI: [body weight]/[height]^2^ (kg/m^2^) and FFMI: [fat-free mass]/[height]^2^ (kg/m^2^).

#### Anaerobic Muscle Fatigue Test

To assess the anaerobic muscular endurance, the Wingate anaerobic test was performed. This test is used to measure the anaerobic capacity and power of an individual (Vandewalle et al., 1987) and has been studied extensively in children and young people (King-Dowling et al., 2018), proving to be a safe tool with good rates of reproducibility (Bar-Or, 1987). A cycle ergometer test was carried out with an individualized load for each athlete, as described before (Bar-Or, 1993). The application of the test allowed us to calculate the minimum, mean, and peak power output as follow: Load (kp) × revolutions in 5 s × 11.765; where the minimum, average and maximum number of revolutions were used for each power measurement (Bar-Or, 2012). Each athlete was constantly consulted for discomfort or pain during the performance of the test through verbal communication.

#### Cardiovascular Parameters

Blood pressure (Omron^®^ Pressure Monitor), systolic blood pressure (SP), and diastolic blood pressure (DP) were measured. The evaluation was carried out with the subject sitting in a chair, allowing to calculating mean arterial pressure (MAP) and pulse pressure (PP). Cardiac autonomic modulation was determined via a recording of RR intervals. The volunteers remained lied on the stretcher in a supine position during the entire HRV measurement procedure, and the RR intervals were continuously recorded during the last 10 min of rest, and 5 min were analyzed. The breathing rate of the subjects were spontaneously. Artifacts and ectopic heartbeats (which did not exceed 3% of the recorded data) were excluded (Task Force of the European Society of Cardiology the North American Society of Pacing Electrophysiology, 1996). The time-domain parameters considered for the analysis were the square root of the mean squared differences of the successive RR intervals (RMSSD, expressed in ms), which reflect the parasympathetic influence (Buchheit et al., 2010) and the standard deviation of the RR intervals (SDNN), which is believed to reflect the total variability, that is, the sympathetic and parasympathetic contribution of the autonomic nervous system on the heart (Berntson et al., 1997; Buchheit and Gindre, 2006). The frequency domains considered were the high frequency (HF) power band that reflects the parasympathetic influence and respiratory sinus arrhythmia (Akselrod et al., 1981) and the low frequency (LF) band associated with baroreflex activity (Goldstein et al., 2011). Very low-frequency band (VLF), which is multi-faceted and is strongly associated with emotional stress (Malik et al., 1996; Fisher et al., 2014; McCraty and Shaffer, 2015). Finally, all the data obtained were digitized and analyzed using the software Kubios HRV^®^ (Tarvainen et al., 2014).

### Statistical Analysis

The report of continuous variables is expressed as mean ± standard deviation (SD) or median and interquartile range (IQR) based on the proximity to the normality of the underlying distribution; categorical variables are presented as absolute (*n*) and relative frequency (%). To verify the assumptions of parametric statistics, we used the *Shapiro-Wilk* test to assess the normality of the distribution of the variables analytically and the *Levene’s* test for homogeneity of variance across groups.

For group comparisons, *Student* t-test (*t*_*Student*_) or *Wilcoxon* rank-sum test (*W*) according to the fulfillment of each of the parametric assumptions. For repeated measures, the *Friedman* rank sum test ($\chi_{Friedman}^{2}$) was used.

For *Student*’s *t*-test, Cohen’s d (*d*_*Cohen*_) was used as a measure of effect size (ES) following the conventions of Cohen (2013), with ES < 0.2 a very small effect, 0.2 ≤ ES < 0.5 small, 0.5 ≤ ES < 0.8 moderate, and ES ≥ 0.8 a large effect. For the *Wilcoxon* signed-rank and rank-sum test, the rank-biserial correlation (${\hat{r}}_{biserial}$) was computed and interpreted according to Funder and Ozer (2019), meaning ES < 0.1 very small, 0.1 ≤ ES < 0.2 small, 0.2 ≤ ES < 0.3 medium, 0.3 ≤ ES < 0.4 large, and ES ≥ 0.4 very large. For each ES, the 95% Confidence Interval (CI_95%_) was also calculated.

A probability of committing a type I (α) error of less than 5%, i.e., a *p* < 0.05, was considered sufficient evidence for statistical significance in hypothesis testing. The statistical analysis was performed using the statistical programming language *R* (version 4.1.0) (R Core Team, 2021) and the statistical packages necessary for this study (Wickham, 2016; Ho et al., 2019; Ben-Shachar et al., 2020; Xie et al., 2020; Castillo Aguilar, 2021; Kassambara, 2021; RStudio Team, 2021).

## Results

### Participants Characterization

**FIGURE 2:**
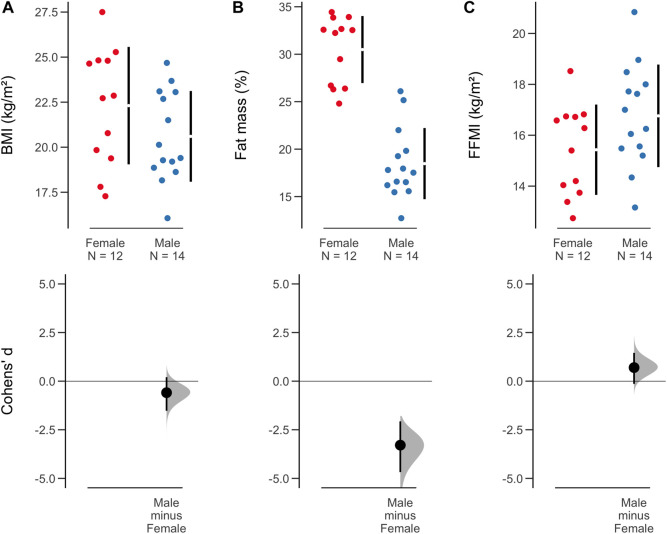
Differences in body composition parameters compared by sex and respective effect sizes. **(A)** Body mass index (BMI); **(B)** fat mass; **(C)** fat-free mass index (FFMI). The dispersion of the sample by sex and the size of the effect through Cohen’s d (ES) are shown. 95% confidence intervals for ES were calculated using bootstrap resampling with 5,000 replications. They are also bias-corrected and accelerated (BCa).

At t-0, we found similar morphological characteristics between males and females, particularly in body weight (*t*_*Student*_ (22) = −0.11, *p* = 0.91, *d*_*Cohen*_ = −0.05, CI_95%_[−0.85, 0.75]), FFMI (*t*_*Student*_ (22) = −1.73, *p* = 0.097, *d*_*Cohen*_ = −0.71, CI_95%_[−1.53, 0.13]), and BMI (*t*_*Student*_ (22) = 1.23, *p* = 0.231, *d*_*Cohen*_ = 0.50, CI_95%_[−0.32, 1.31]). As expected, the groups were not homogeneous in all morphological measures at baseline, as males were taller on average (1.65 ± 0.08 m) than females (1.58 ± 0.03 m; *t*_*Student*_ (24) = −2.95, *p* = 0.007, *d*_*Cohen*_ = −1.16, CI_95%_[−1.99, −0.31]). Similarly, females had a higher body fat (30.2 ± 3.5%) than their male counterparts (18.3 ± 3.4%; *t*_*Student*_ (22) = 8.44, *p* < 0.001). However, when comparing BMI or FFMI, we didn’t find any statistically significant difference between sexes (BMI, *t*_*Student*_ (22) = 1.23, *p* = 0.231; FFMI, *t*_*Student*_ (22) = −1.73, *p* = 0.097). Estimates of the magnitude of sex differences in BMI, body fat, and FFMI, using the mean value for each period for each participant, are shown in Figure 2.

In cardiac parameters, a within-participants analysis by sex shows variations in systolic pressure among females ($\chi_{Friedman}^{2}$ (4) = 18.60, *p* < 0.001, *W*_*Kendall*_ = 0.37, CI_95%_[0.26, 0.63]), whereas in males these values remained constant over time periods ($\chi_{Friedman}^{2}$ (4) = 2.06, *p* = 0.725, *W*_*Kendall*_ = 0.05, CI_95%_[0.02, 0.46]). The same pattern was seen in mean arterial pressure (females, $\chi_{Friedman}^{2}$ (4) = 14.92, *p* = 0.005, *W*_*Kendall*_ = 0.29, CI_95%_[0.13, 0.58]; males, $\chi_{Friedman}^{2}$ (4) = 8.94, *p* = 0.063, *W*_*Kendall*_ = 0.23, CI_95%_[0.10, 0.67]). Regarding the blood pressure parameters, although the PP parameters were consistently higher in males (50.5 (23.5) mmHg vs females, 37.5 (11) mmHg), there was only a significant difference in the parameters at t-0 (*W* = 34, *p* = 0.03, ${\hat{r}}_{biserial}$ = −0.53, CI_95%_[−0.79, −0.12]) when comparing both groups.

A similar situation was observed when analyzing the anaerobic muscle fatigue and power parameters, where males registered higher values than females, but only at t-0 (males, 402.8 ± 125 W; females, 304.7 ± 108.3 W), *t*_*Student*_ (23) = −2.09, *p* = 0.048, *d*_*Cohen*_ = −0.84, CI_95%_[−1.65, −0.01]. All values for blood pressure and anaerobic muscle fatigue test results are presented in Table 1.

**TABLE 1:** Blood pressure and anaerobic muscle fatigue test results are presented for each of the time periods separated by sex.

	Female					Male				
	t-0	t-1	t-2	t-3	t-4	t-0	t-1	t-2	t-3	t-4
	*n* = 12	*n* = 12	*n* = 12	*n* = 12	*n* = 12	*n* = 12	*n* = 14	*n* = 12	*n* = 12	*n* = 11
SP^np^ (mmHg)	104.5−(7.2)	115−(11.0)^La^	111−(7.0)^La^	111−(7.0)^La^	107.5−(7.2)	118−(14.5)	114−12.8	115−(13.8)	111−(10.5)	114−(16)
DP^np^ (mmHg)	67−(5.8)	72−6.2	69−(4.0)^La^	69−(4.0)^La^	68−(9)	62−(8.2)	69−17.5	66−(9.8)	66−(13.5)	63−(9.5)
MAP^np^ (mmHg)	78.3−(8.4)	85.5−(3.4)^La^	82.5−(8.2)^La^	82.5−(7.4)^La^	80.8−(7)	79.5−(9.5)	85.3−10.5	84.8−(11)	82.5−(10.3)	81.7−(8.8)
PP^np^ (mmHg)	37.5−(11)	46.5−(10.0)^La^	43−(7.2)^La^	43−(7.2)^La^	42.5−(6.8)	50.5−(23.5)	44.5−16.2	51.5−(10)	45.5−(17.5)	46−(10.5)
PPO^p^ (W)	304.7 ± 108.3	243.9 ± 90.0^La^	269.5 ± 90.6^Mb^	274.8 ± 98.3^La^	297.1 ± 85.2^Mcd^	402.8 ± 125	310.7 ± 134.9^La^	341.3 ± 151.9^La–Mb^	361.5 ± 130.6^Lb^	372 ± 135
MePO^p^ (W)	284.2 ± 88.8	224.4 ± 79.2^La^	239 ± 75.6	240.9 ± 90.3^La^	266.4 ± 70.5^*Ld*^	368.1 ± 119.2	295.9 ± 125.5^La^	303 ± 138.3^La^	325.8 ± 118.4^Lab^	336.6 ± 105.5^Mc^
MiPO^p^ (W)	236.8 ± 71.7	181.3 ± 64.1^La^	181.8 ± 57.6^Ma^	206.8 ± 73.6	228 ± 68.2^*Lc*^	301.8 ± 90.4	238.6 ± 102.2^La^	223.5 ± 94.0^La^	268.9 ± 100.4^La^	273.8 ± 88.3^Mc^
Fatigue^p^ (%)	20.8 ± 10	24.3 ± 9.5	30.4 ± 11.8^Ma^	24.2 ± 9	23 ± 13.6	27.1 ± 11	23.9 ± 10.8	33.7 ± 20.2	23.2 ± 13.3	25.6 ± 11

*Values are presented as median−(IQR) or mean ± SD as appropriate. All pairwise comparison was performed using Wilcoxon signed-rank test (np) or Student t-test (p) and for effect-size (rank-biserial correlation or Cohens’d) codes were: S, small; M, moderate; L, large. For blood pressure code: SP, systolic pressure; DP, diastolic pressure; MAP, mean arterial pressure; PP, pulse pressure. For Wingate test parameters: PPO, peak power output; MePO, mean power output; MiPO, minimum power output. The differences within the same group indicate a p < 0.05, following the code: a, compared with t-0; b, compared with t-1; c, compared with t-2; d, compared with t-3; e, compared with t-4.*

**FIGURE 3:**
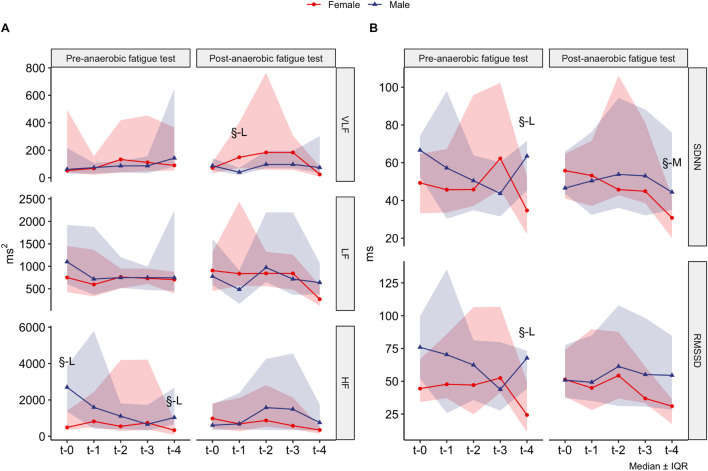
Heart rate variability parameters were compared by sex within each time. **(A)** Frequency domain; **(B)** time domain. The median of the values is presented in a red line for the females and blue for the males, while the red and blue areas represent the corresponding interquartile range. § Significantly different (*p* < 0.05) and rank-biserial correlation code were: M, moderate; L, large.

When analyzing HRV parameters, we observed longitudinal variations only in the VLF-post anaerobic fatigue test frequency domain ($\chi_{Friedman}^{2}$ (4) = 13.16, *p* = 0.011, *W*_*Kendall*_ = 0.14, CI_95%_[0.07, 0.31]), while the other autonomic-cardiac parameters remained constant when not considering sex as an influencing factor. However, after separating our analyses by sex, we found that this relationship with VLF-post is valid only for males ($\chi_{Friedman}^{2}$ (4) = 11.64, *p* = 0.02, *W*_*Kendall*_ = 0.22, CI_95%_[0.16, 0.46]), but not for females ($\chi_{Friedman}^{2}$ (4) = 9.24, *p* = 0.055, *W*_*Kendall*_ = 0.21, CI_95%_[0.11, 0.53]). When comparing directly between sexes, we observed that females had a higher VLF-post anaerobic fatigue test than males at t-1, while at HF-pre anaerobic fatigue test was higher in males than females at t-0. In the time domain, females reached lower levels of SDNN, both pre and post-anaerobic fatigue test at t-4, but not in the other periods. Similarly, we found large differences between males and females in RMSSD pre- but not post-anaerobic fatigue test (see Figure 3).

## Discussion

Our study aimed to evaluate the differences in the cardiac autonomic modulation of young competitive swimmers through non-invasive measures and in response to muscle fatigue caused by high-intensity exercise during two consecutive competition periods. Our findings in the cardiac parameters show that the male had a significant parasympathetic predominance (denoting major cardiac autonomic modulation), evidenced in the difference in HF-pre compared to females in t-0 and t-4. Although the HF-pre values progressively decreased in males, it remains higher than the values registered in females. We believe that these differences could be due to two factors. The first is caused by non-modifiable variables (age and sex), which can be strong predictors of post-exercise cardiac recovery in young (Jezdimirovic et al., 2017); The second factor can be given by body fat mass. It has been observed in prepubertal children in Europe that a higher amount of body fat (31.4% in males and 37.3% in females) is related to a decrease in parasympathetic modulation (Santos-Magalhaes et al., 2015), which coincidentally resembles the values evaluated in competitive swimmers in our study (30.3 ± 3.5% fat mass). Similarly, elevated body fat levels have been linked to altered states in cardiac autonomic activity after a 20-min jog event (Santana et al., 2019), considering that the delay in autonomic recovery after exercise has been associated with an increased risk of cardiovascular events (Albert et al., 2000; Rossi et al., 2015). These would indicate that many genetic, sociocultural, lifestyle, and morphological variables may directly impact the baseline response of cardiac autonomic modulation (Delgado-Floody et al., 2020).

Concerning the pre and post-anaerobic fatigue test, at t-1, both sexes followed a similar pattern in terms of anaerobic power records compared to the muscle fatigue test. In t-1, the different powers decrease compared to t-0, to then increase progressively to t-4, which would be explained by the training and progressive tapering of sports planning (Le Meur et al., 2012; Vachon et al., 2021). Before the anaerobic test, the difference in the autonomic parameters between the groups decreased progressively until t-3, where the frequency domain values are practically unified. This decrease in cardiac autonomic control is observed before the second competition, neurophysiologically showing the autonomic wear of the swimmers in the face of an anaerobic fatigue test (Schneider et al., 2019). However, in t-4, the groups again differentiate, showing greater cardiac autonomic and especially parasympathetic reactivity on the part of males in both RMSSD-post and SDNN-post. After the anaerobic muscle fatigue test, the cardiac autonomic response shows that females at t-2 show greater changes at VLF-post, which tend to remain in time until t-3. It is known that the cardiac autonomic recovery of swimmers differs in terms of the type of test they perform. In more extended tests, the recovery is faster, while in speed tests, the recovery is slower (Piras et al., 2019). This difference could be affecting the females in our study more, given her response to emotional stress in relation to the evaluations carried out in this study. In this sense, it is noteworthy that increased effort intensity supplants the female’s advantage in resistance to fatigue (Spina et al., 2019).

In the case of the males, they progressively show greater post-test cardiac autonomic modulation, which becomes different from females in SDNN-post during t-4. This difference may be due to a better cardiac adaptation to training, represented by a vagal predominance in cardiac autonomic modulation (Triposkiadis et al., 2002), which could be better in males than in females due to their different morphological characteristics (Bassareo and Crisafulli, 2020; Hottenrott et al., 2021).

Regarding morphological parameters, a similar character was evidenced in the participants. However, we observed that at all times, females registered higher levels of body fat than males. Although this is expected, other studies have linked a high percentage of body fat with low sympathetic activity (Rossi et al., 2014) and decreased parasympathetic modulation in time-domain HRV (Santos-Magalhaes et al., 2015). Therefore, these antecedents could help understand the differences in the neurophysiological response to the stress of exercise of young swimmers. However, these should be studied in depth in future studies in the area.

## Limitations

The main limitations of this study were the small sample size and the non-probabilistic selection of the participants, which could limit the external validity of the study. In addition, for future research, we suggest controlling the hours of rest and sleep of swimmers, which could be factors that influence the physical and physiological performance of athletes. We also believe that it would be appropriate to be able to use an anaerobic muscle fatigue test carried out in the aquatic environment, which could better assimilate the muscle fatigue that athletes perform under competition conditions. Despite this, our study reports novel information on how the autonomic response varies during consecutive competition periods of young swimmers, which has been little studied and could affect their neurophysiological response in competition.

## Conclusion

There are differences in the cardiac autonomic modulation of young swimmers during consecutive periods of competition. Males show greater parasympathetic reactivity after the anaerobic muscle fatigue test after two consecutive competition periods, while females show less cardiac autonomic modulation during the same competition periods.

## Data Availability Statement

The raw data supporting the conclusions of this article will be made available by the authors, without undue reservation.

## Ethics Statement

The studies involving human participants were reviewed and approved by The Ethics Committee of the University of Magallanes, Chile (N°141CEC2018). Written informed consent to participate in this study was provided by the participants’ legal guardian/next of kin.

## Author Contributions

All authors listed have made a substantial, direct and intellectual contribution to the work, and approved it for publication.

## Conflict of Interest

The authors declare that the research was conducted in the absence of any commercial or financial relationships that could be construed as a potential conflict of interest.

## Publisher’s Note

All claims expressed in this article are solely those of the authors and do not necessarily represent those of their affiliated organizations, or those of the publisher, the editors and the reviewers. Any product that may be evaluated in this article, or claim that may be made by its manufacturer, is not guaranteed or endorsed by the publisher.
